# Plasmid content of carbapenem resistant *Acinetobacter baumannii* isolates belonging to five International Clones collected from hospitals of Alexandria, Egypt

**DOI:** 10.3389/fcimb.2023.1332736

**Published:** 2024-01-09

**Authors:** Sandra Sánchez-Urtaza, Alain Ocampo-Sosa, Jorge Rodríguez-Grande, Mohammed A. El-Kholy, Sherine M. Shawky, Itziar Alkorta, Lucia Gallego

**Affiliations:** ^1^ Laboratory of Antibiotics and Molecular Bacteriology, Department of Immunology, Microbiology and Parasitology, Faculty of Medicine and Nursing, University of the Basque Country, Leioa, Spain; ^2^ Microbiology Service, University Hospital Marqués de Valdecilla, Health Research Institute (IDIVAL), Santander, Spain; ^3^ CIBER de Enfermedades Infecciosas (CIBERINFEC), Instituto de Salud Carlos III, Madrid, Spain; ^4^ Department of Microbiology and Biotechnology, Division of Clinical and Biological Sciences, College of Pharmacy, Arab Academy for Science, Technology & Maritime Transport (AASTMT), Alexandria, Egypt; ^5^ Medical Research Institute, Alexandria University, Alexandria, Egypt; ^6^ Department of Biochemistry and Molecular Biology, Faculty of Science and Technology, University of the Basque Country, Leioa, Spain

**Keywords:** *Acinetobacter baumannii*, plasmids, replicon typing, antibiotic resistance genes, whole genome sequencing

## Abstract

Multidrug resistant *Acinetobacter baumannii* is one of the most important nosocomial pathogens worldwide. During the last decades it has become a major threat for healthcare settings due to the high antibiotic resistance rates among these isolates. Many resistance determinants are coded by conjugative or mobilizable plasmids, facilitating their dissemination. The majority of plasmids harbored by *Acinetobacter* species are less than 20 Kb, however, high molecular weight elements are the most clinically relevant since they usually contain antibiotic resistance genes. The aim of this work was to describe, classify and determine the genetic content of plasmids harbored by carbapem resistant *A. baumannii* isolates belonging to predominant clonal lineages circulating in hospitals from Alexandria, Egypt. The isolates were subjected to S1-Pulsed Field Gel Electrophoresis experiments to identify high molecular weight plasmids. To further analyze the plasmid content and the genetic localization of the antibiotic resistance genes, isolates were sequenced by Illumina Miseq and MinION Mk1C and a hybrid assembly was performed using Unicycler v0.5.0. Plasmids were detected with MOBsuite 3.0.3 and Copla.py v.1.0 and mapped using the online software Proksee.ca. Replicase genes were further analyzed through a BLAST against the *Acinetobacter* Plasmid Typing database. Eleven plasmids ranging in size from 4.9 to 205.6 Kb were characterized and mapped. All isolates contained plasmids, and, in many cases, more than two elements were identified. Antimicrobial resistance genes such as *bla*_OXA-23_, *bla*_GES-like_, *aph(3’)-VI* and *qacEΔ1* were found in likely conjugative large plasmids; while virulence determinants such as septicolysin or TonB-dependent receptors were identified in plasmids of small size. Some of these resistance determinants were, in turn, located within transposons and class 1 integrons. Among the identified plasmids, the majority encoded proteins belonging to the Rep_3 family, but replicases of the RepPriCT_1 (Aci6) family were also identified. Plasmids are of high interest as antibiotic resistance control tools, since they may be used as genetic markers for antibiotic resistance and virulence, and also as targets for the development of compounds that can inhibit transfer processes.

## 1 Introduction

Carbapenem resistant *Acinetobacter baumannii* is a major global concern for healthcare settings and, according to the World Health Organization, it is considered one of the critical pathogens for which new antibiotics are urgently needed (Tacconelli et al., 2018; Vázquez-López et al., 2020). The high rates of antibiotic resistance among clinical isolates are the main cause of fatal nosocomial infections including: wound infections, ventilator-associated pneumonia and other respiratory tract infections, meningitis, bacteremia, bloodstream infections and urinary tract infections (Almasaudi, 2018; Ayoub Moubareck and Hammoudi Halat, 2020).

The Mediterranean Area is one of the hotspots for the emergence and dissemination of antibiotic resistance genes (Ministry for Health and Ministry for Agriculture, Fisheries & Animal Rights, 2020). In Southern-Europe, countries such as Croatia, Greece, Cyprus and Italy show high rates of combined antibiotic resistance rates to fluoroquinolones, aminoglycosides and carbapenems being 98.5%, 91.4%, 88.8% and 84.7%, respectively (European Centre for Disease Prevention and Control, (ECDC), 2021). In countries of the Middle East and North Africa, antibiotic resistance rates are on the rise, mainly due to the lack of effective infection control measures and the abusive use of antibiotics (Higgins et al., 2021). As an example, prevalence of multidrug resistant *A. baumannii* in Egypt ranges from 30 to 100%, and between 26.6% and 100% of the isolates are carbapenem resistant (El-Kholy et al., 2021).

The spread of multi-drug resistant isolates can be facilitated by mobile genetic elements (MGE) such as plasmids. Plasmids are extrachromosomal DNA molecules with their own replication mechanism that do not encode essential genes required by the host, but usually contain genes conferring advantages in certain environmental conditions, such as antimicrobial resistance genes (Partridge Sally et al., 2018). Plasmids harbored by *Acinetobacter* spp. vary in size from 1.3 Kb to 400 Kb but the majority are less than 20 Kb (Brovedan et al., 2020). However, high molecular weight plasmids are the most clinically relevant, as they usually contain a wide variety of antibiotic resistance genes and mobile genetic elements such as integrons or transposons (Brovedan et al., 2020).

One of the most frequently used strategies to categorize plasmids is the identification and classification of their replication initiator proteins (Rep). To date, two schemes have been described for this purpose. *Acinetobacter baumannii* PCR-Based Replicon Typing (AB-PBRT) was first proposed and it was able to identify 27 replicase genes grouped in 19 homology groups (GR1-GR19) (Bertini et al., 2010). However, since its publication, more replicase genes have been identified which were not identifiable by this method. Thus, in 2022, Lam et al. developed a whole genome sequencing based database including 621 plasmid sequences organized in four groups based on their replicases: Rep_1, RepPriCT_1, Rep_3 and a group lacking an identifiable Rep protein (Lam et al., 2022). It is worth mentioning that those plasmids with the same Rep protein are unable to coexist stably in the same cell, which is called plasmid incompatibility (Brovedan et al., 2020). Other plasmid classification methods using bioinformatic tools have also been developed, such as COPLA, that allow species-independent and universal plasmid classification (Redondo-Salvo et al., 2021).

Most plasmids around 20 Kb that are frequently found in the genus belong to the Rep_3 family or the pRAY derivative plasmids (Brovedan et al., 2020). Those belonging to the first group, contain partition systems, type II toxin-antitoxin systems, relaxase gene, virulence genes (TonB-dependent receptors and septicolysin) and antibiotic resistance genes (*bla*_OXA-23_, *bla*_OXA-58_, *bla*_OXA-72_ and *tet(39))*. Some of these plasmids can also harbor genes coding for mobilization proteins such as MobA and MobL, which are important for their mobilization through other plasmids and can also be used for plasmid classification (Alvarado et al., 2012). Moreover, a group of small plasmids belonging to the Rep_1 superfamily have been identified, these plasmids contain a *rep* gene and two to five genes coding for hypothetical proteins with unknown function (Lean and Yeo, 2017; Partridge Sally et al., 2018; Brovedan et al., 2020).

High molecular weight plasmids are less frequent, they are the most clinically relevant as they usually contain carbapenem and aminoglycoside resistance genes such as *bla*_OXA-23_ or *aphA6* (*aph(3′)-VIa*), and usually belong to the homology group GR6 following Bertini scheme (Partridge Sally et al., 2018; Brovedan et al., 2020). Other high molecular weight plasmids found in *A. baumannii* and other species of the genus are plasmid pNDM-BJ01 and its derivatives, which contain the *bla*_NDM-1_ gene within transposon Tn*125*, and the *aphA6* gene with IS*Aba4* upstream of the gene (Partridge Sally et al., 2018; Brovedan et al., 2020).

As these elements may be transferred to other bacteria by conjugative processes, they are one of the main causes of the dissemination of antibiotic resistance (Partridge Sally et al., 2018; Jeon et al., 2023). Conjugation is an important mechanism used by some plasmids to share genes between bacterial strains or species through a conjugative pilus (Mindlin et al., 2021). Additionally, some of these conjugative plasmids can help in mobilization of non-conjugative mobilizable plasmids (Brovedan et al., 2020). Co-existence of self-transmissible and mobilizable plasmids in the same cell is a serious threat for transmission of antibiotic resistance and virulence determinants.

Currently, there is little information on the circulating plasmids in Egypt. Since this country is one of the gateways for resistance genes and plasmids dissemination from the Middle East to other countries of the Mediterranean Area, more studies are needed for a better understanding and control of the situation. Thus, the aim of this work was to describe, classify and determine the genetic content of plasmids harbored by carbapenem resistant *A. baumannii* isolates belonging to predominant clonal lineages circulating in hospitals from Alexandria, Egypt.

## 2 Materials and methods

### 2.1 Bacterial isolates

Six carbapenem resistant *Acinetobacter baumannii* clinical isolates were selected from a group of 36 isolates collected in hospitals from Alexandria, Egypt. These isolates were previously characterized by the analysis of plasmid profiles obtained by conventional lysis, PCR-Based Replicon Typing to identify the replicases, and by Multi Locus Sequence Typing experiments to determine their clonal lineage (Sánchez-Urtaza et al., 2023). We made the selection of these isolates based on the different replicase combinations we found and the International Clone they belonged to, choosing an isolate that represents each group: Ale9 (IC8; Aci1/2 + p2S1), Ale10 (IC2; Aci6 + Aci8/9), Ale18 (IC9; Aci6), Ale20 (IC2; Aci1/2 + Aci6), Ale21 (IC4; Aci4 + Aci8/9) and Ale26 (IC5; Aci6 + p2S1). The main characteristics of the isolates are showed in **Table 1**.

**Table 1:** Characteristics of the *A. baumannii* isolates studied in this work.

Isolate	International Clone	Pasteur ST	β-lactamase genes	Other antibiotic resistance genes
**Ale9**	IC8	613	*bla*_ADC-211_ *, bla*_PER-7_ *, bla*_OXA-23_ *, bla*_OXA-68_	*cmlA5, arr-2, mph(E), msr(E), armA, strA, strB, aph(3´)-VI, ant(3’’)-IIa, sul1, sul2, tet(B), tet(39)*
**Ale10**	IC2	2	*bla*_ADC-143_ *, bla*_PER-7_ *, bla*_OXA-23_ *, bla*_OXA-66_	*catB8, mph(E), msr(E), armA, strA, strB, aph(3´)-Ia, aac(6’)-Ib, ant(3’’)-II, aadA1-pm, sul1, sul2*
**Ale18**	IC9	85	*bla*_ADC-80_ *, bla*_GES-11_ *, bla*_OXA-23_ *, bla*_OXA-94_	*aph(3´)-VI, aac(6’)-Ib, ant(3’’)-II, sul1, dfrA7*
**Ale20**	IC2	570	*bla*_TEM-1_ *, bla*_ADC-25_ *, bla*_NDM-1_ *, bla*_OXA-23_ *, bla*_OXA-66_	*catB8, mph(E), msr(E), armA, aph(3´)-VI, aac(6’)-Ib, ant(3’’)-II, aadA1-pm, sul1*
**Ale21**	IC4	15	*bla*_ADC-263_ *, bla*_OXA-23_ *, bla*_OXA-51_	*cmlA5, arr-2, mph(E), msr(E)* *armA, strA, strB, aph(3’)-VIa, ant(3’’)-II, sul1*
**Ale26**	IC5	158	*bla*_ADC-117_ *, bla*_GES-35_ *, bla*_OXA-23_ *, bla*_OXA-65_	*catB8, mph(E), msr(E), armA, aph(3´)-Ia, aph(3´)-VIa, ant(3’’)-II, aadA1-pm, sul1*

### 2.2 S1-Pulsed Field Gel Electrophoresis

To investigate the presence of high molecular weight plasmids S1-Pulsed-Field Gel Electrophoresis (PFGE) experiments were performed as follows. Briefly, 500 µL of each bacterial culture in Mueller Hinton broth at a concentration of 3 McFarland were mixed with 500 µL of low-melting agarose (2% in TE pH 8) within a hot water bath at 50°C. Reusable plug molds were filled with this mixture and plugs were allowed to solidify for 15 minutes at 4°C. The resulting plugs were treated with lysis solutions including lysozyme (50 mg/mL) and proteinase K (20 mg/mL) to extract DNA. The Bacterial DNA embedded in low-melting agarose plugs was digested using 14 units of S1-nuclease (Takara Bio, Kusatsu, Japan) per plug and then it was run on a CHEF-DR III system (Bio-Rad, Munich, Germany) for 17 h at 6 V/cm, switch time of 5-35 s and 14°C. As molecular weight marker, CHEF DNA Size Standard, 48.5-1,000 Kb, Lambda Ladder (Bio-Rad) was used.

### 2.3 Whole Genome Sequencing (WGS) experiments

The isolates were sequenced by both Illumina and Oxford Nanopore methodology as previously described (Sánchez-Urtaza et al., 2023). Total DNA was extracted using Qiagen DNeasy^®^ Blood and Tissue (Qiagen, Hilden, Germany), according to the protocol supplied by the manufacturer. The quality of the DNA was determined by measuring concentration and purity using a spectrophotometer NanoDrop™ 1000 (Thermo Fisher Scientific, Waltham, MA, USA). DNA purity was estimated by calculating the A260/A280 ratios. Samples with ratios between 1.8-2.0 were assumed to be pure. In addition, DNA concentration was measured with the Qubit 4 Fluorimeter (Thermo Fisher Scientific) using the 1X dsDNA high‐sensitivity assays kit (Invitrogen). Sequencing experiments were carried out on a MiSeq device using reagents kit v3 for 2×300 paired-end libraries (Illumina Inc., San Diego, CA, USA), and on a MinION Mk1C device with the Rapid Barcoding Kit (SQK-RBK004) in an R9 flow cell (FLO-MIN106) (Oxford Nanopore Technologies, Cambridge, UK).Basecalling and barcoding of Oxford Nanopore Technology reads was carried out using guppy v.6.5.7+ca6d6af and minimap2 version 2.24-r112, using super-quality mode. Filtering of reads shorter than 250 bp or with average quality less than Q10 and quality checks were performed with NanoPack v.1.4.1 (De Coster and Rademakers, 2023). Read contamination was checked by the use of Kraken v.2.1.2 8 (Wood et al., 2019). The reads were assembled with Flye v2.9.2-b1786 (Kolmogorov et al., 2019) and polished using medaka v.1.7.2 (https://github.com/nanoporetech/medaka). Assembly completion was tested visually using Bandage (Wick et al., 2015) and quality was checked out using Quast v.5.2.0 (Gurevich et al., 2013). Genome sequences of the isolates were generated by combining data from both the Illumina and MinION data sets using Unicycler v0.5.0 (https://github.com/rrwick/Unicycler). Then, assemblies were annotated using Bakta v.1.7.0 (Schwengers et al., 2021), and Abricate v1.0.1 (https://github.com/tseemann/abricate) was used to find antimicrobial resistance genes in the following databases: NCBI-AMRFinderPlus (Feldgarden et al., 2019), CARD (Jia et al., 2016), ResFinder (Zankari et al., 2012) and ARG-ANNOT (Gupta et al., 2014). Plasmids were detected using MOBsuite:3.0.3 (Robertson and Nash, 2018) and Copla.py v.1.0 (Redondo-Salvo et al., 2021) and were mapped with Proksee.ca (Grant et al., 2023). Plasmid sequences alignments were performed using the FastANI 1.3.3 software (Jain et al., 2018) integrated in Proksee.ca. Finally, *Acinetobacter* Plasmid Typing database against the draft genome was used to determine the presence of replicase genes and classify plasmids as previously described (Lam et al., 2022). The whole-genome shotgun sequences of the isolates generated for this study were deposited and can be found in GenBank under the BioProject accession number PRJNA856145. The versions of the genomes described in this paper are versions JARNLX020000000, JARNLW020000000, JARNLO020000000, JARNLM020000000, JARNLL020000000 and JARNLH020000000.

### 2.4 Conjugation experiments

Filter mating experiments were carried out in order to know whether betalactamase/carbapenemase genes were located in conjugative plasmids. Briefly, flasks containing 20 mL of Mueller Hinton broth (MHB) were inoculated independently with single colonies of donor strains of *A. baumannii* and recipient strain *Escherichia coli* J53, sodium azide-resistant and incubated overnight with appropriate antibiotics at 37°C and 200 rpm. The next day, cultures were used to inoculate fresh flasks with 20 mL MHB until they reached an OD600 of 0.6. Cultures were then harvested and washed with MHB to remove traces of antibiotics, mixed at equal ratios (1:1) and pelleted by centrifugation. The pellet was resuspended in 200 μL of MHB and subjected to filter mating on MH agar plates. Conjugation plates were incubated overnight at 37°C. Transconjugants were selected onto MH agar plates supplemented with corresponding antibiotics (ceftazidime-8 µg/mL, meropenem-4 µg/mL) and sodium azide (200 µg/mL). Transconjugants were confirmed by carbapenemase multiplex PCR (Cerezales et al., 2021).

## 3 Results

### 3.1 S1-Pulsed Field Gel Electrophoresis

**Figure 1:**
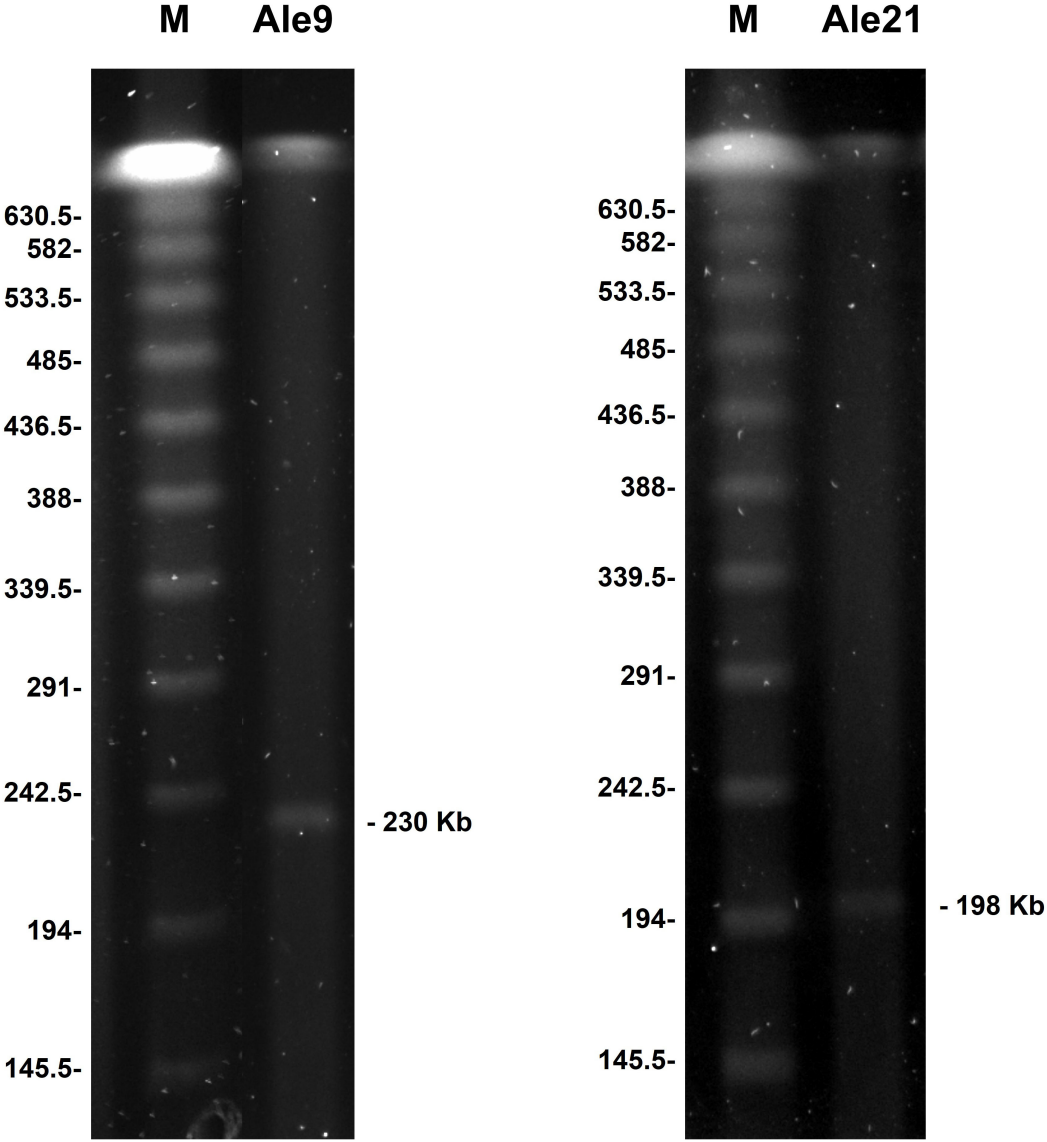
High molecular weight structures identified by S1-PFGE. Sizes are indicated in Kb. M: molecular weight marker CHEF DNA Size Standard, 48.5-1,000 Kb, Lambda Ladder.

Plasmid analysis by S1-PFGE experiments revealed the presence of high molecular weight plasmids of approximately 230 and 197 Kb in isolates Ale9 and Ale21, respectively (**Figure 1**). No plasmids were detected using this technique in the rest of the isolates.

### 3.2 Plasmid mapping

Through sequencing experiments and hybrid assembly, 11 plasmids were identified and classified according to their size and replicase type, and their specific structures were determined (**Table 2**).

**Table 2:** Characterized plasmids by whole genome sequencing and hybrid assembly, and replicases identified by AB-PBRT and *Acinetobacter* Plasmid Typing database methods (>95% identity).

Isolate	International Clone	Plasmid size (Kb)	Plasmid name	G+C content (%)	Predicted transferability	AB-PBRT Bertini	*Acinetobacter* Plasmid Typing database replicases
**Ale9**	IC8	205.657	pAbAle9	40.8	Conjugative	-	Rep_3 family protein R3-T60
**Ale10**	IC2	70.016	pAbAle10.1	33.5	Conjugative	RepAci6	RepPriCT_1 family protein RP-T1
		10.931	pAbAle10.2	34.9	Mobilizable	RepAci9	Rep_3 family protein R3 -T4
**Ale18**	IC9	80.303	pAbAle18	35.7	Conjugative	RepAci6	RepPriCT_1 family protein RP-T1
**Ale20**	IC2	88.488	pAbAle20	33.6	Conjugative	RepAci6/RepAci1	RepPriCT_1 family protein RP-T1/Rep_3 family protein R3 -T1
**Ale21**	IC4	199.267	pAbAle21.1	38.1	Conjugative	-	Rep_3 family protein R3-T60/Rep_3 family protein R3-T13
		14.246	pAbAle21.2	39.2	Mobilizable	-	-
		8.771	pAbAle21.3	37.7	Mobilizable	-	Rep_3 family protein R3-T14
		5.234	pAbAle21.4	34	Non-mobilizable	-	-
**Ale26**	IC5	96.763	pAbAle26.1	35.2	Conjugative	RepAci6	RepPriCT_1 family protein RP-T1
		4.955	pAbAle26.2	40.2	Mobilizable	-	Rep_3 family protein R3-T15

"-": no replicases detected by this method.

**Figure 2:**
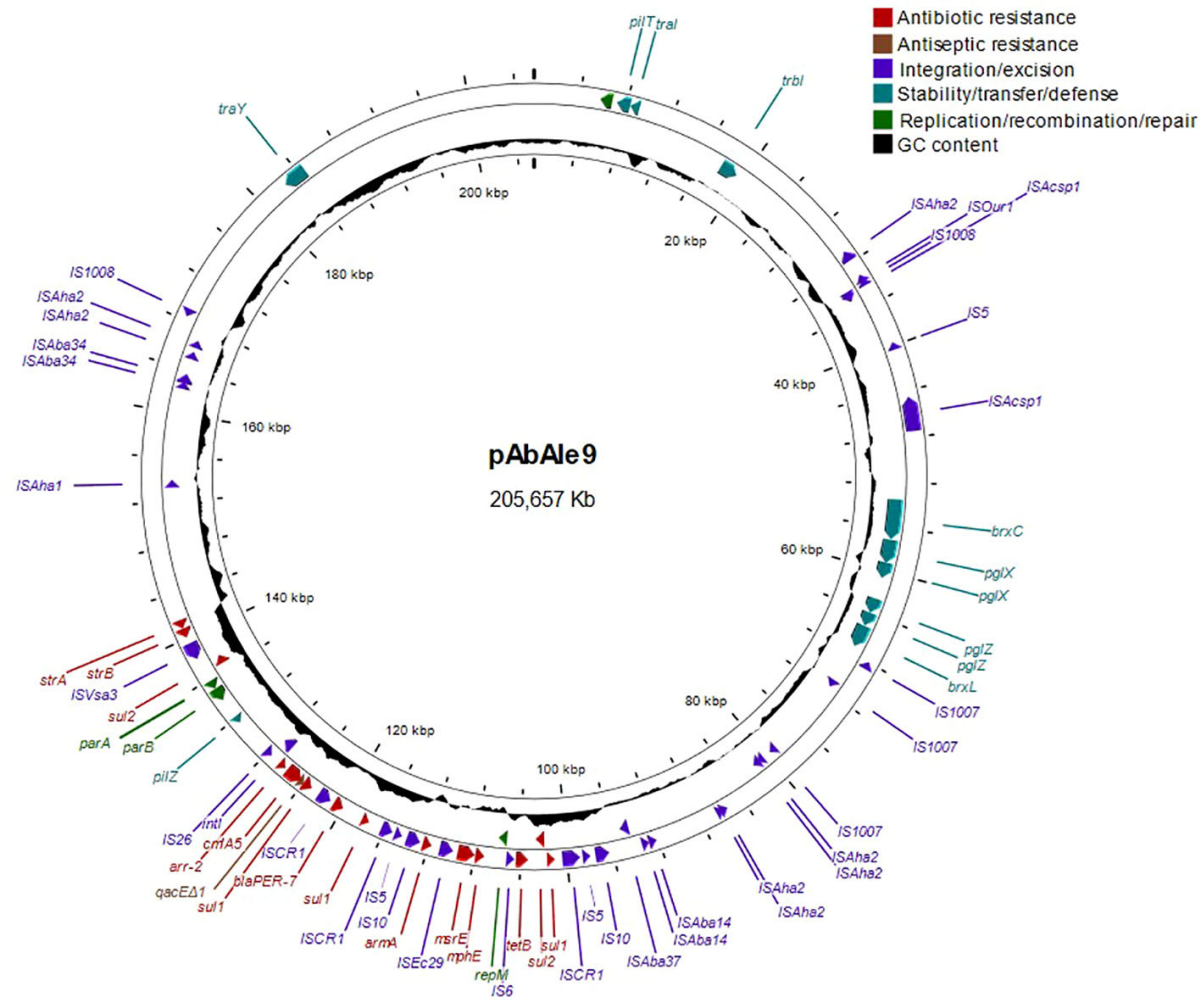
Structure of large plasmid pAbAle9 identified by whole genome sequencing in isolate Ale9.

A likely conjugative large plasmid of 205.657 Kb was identified in isolate Ale9, which contained the *bla*_PER-7_, *sul1*, *sul2*, *tet(B)*, *mph(E)*, *msr(E)*, *cmlA5*, *arr-2*, *strA*, *armA*, *strB* and genes *qacEΔ1* (**Figure 2**). The rest of the genes were chromosome-borne (*bla*_ADC-211_, *bla*_OXA-23_, *bla*_OXA-68_ *, aph(3´)-VI, ant(3’’)-IIa* and *tet(39)*).

**Figure 3:**
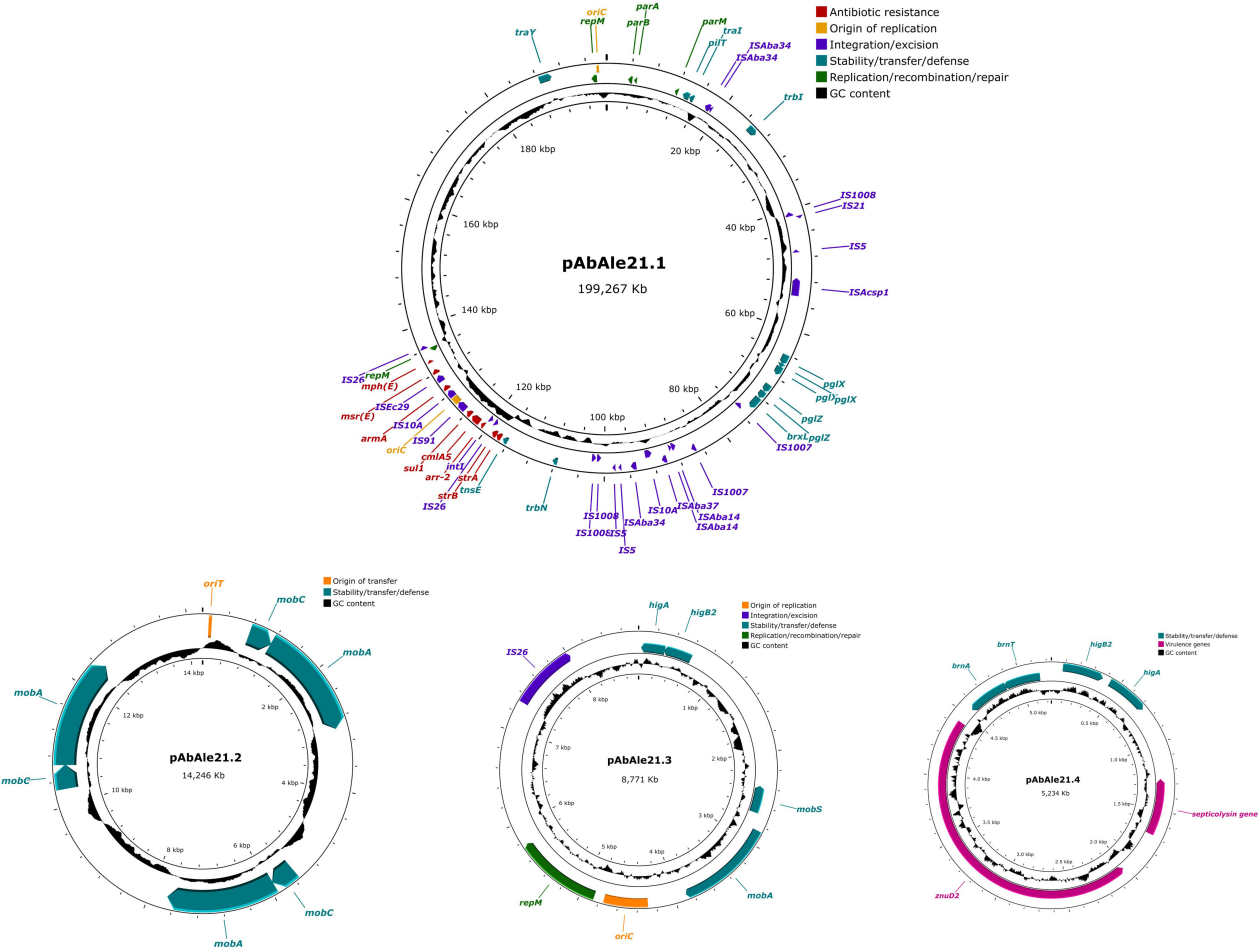
Structure of plasmid pAbAle21.1 and low molecular weight plasmids pAbAle21.2, pAbAle21.3 and pAbAle21.4 identified by whole genome sequencing in isolate Ale21.

In isolate Ale21 a large plasmid of 199.267 Kb was also discovered together with two mobilizable plasmids of 14.246 Kb and 8.771 Kb, and a non-mobilizable plasmid of 5.234 Kb (**Figure 3**). Some antibiotic resistance genes were found in plasmid pAbAle21.1 (*armA*, *strA*, *strB*, *sul1*, *mph(E)*, *msr(E)*, *arr-2* and *cmlA5*). Genes coding for mobilization proteins such as *mobA*, *mobC* y *mobS*, were identified in plasmids pAbAle21.2 and pAbAle21.3. Additionally, genes coding for virulence factors such as TonB-dependent receptors and septicolysin were discovered in plasmid pAbAle21.4. The rest of the antibiotic resistance genes were chromosome-borne (*bla*_ADC-263_, *bla*_OXA-23_, *bla*_OXA-51_, *aph(3’)-VI* and *ant(3’’)-II*). The hybrid assembly also discovered a copy of a *bla*_OXA-23_ variant within the chromosome, the *bla*_OXA-166_. As it is shown in **Supplementary Figure 1**, it is worth mentioning that plasmids pAbAle9 and pAbAle21.1 are phylogenetically related (Average Nucleotide Identity 99.52%) (**Supplementary Figure 1**), sharing replicons (Rep_3 family protein R3-T60), some antimicrobial resistance genes (*cmlA5, arr-2, mph(E), msr(E), armA, strA, strB, sul1*) and probably the conjugative Type 4 secretion systems.

**Figure 4:**
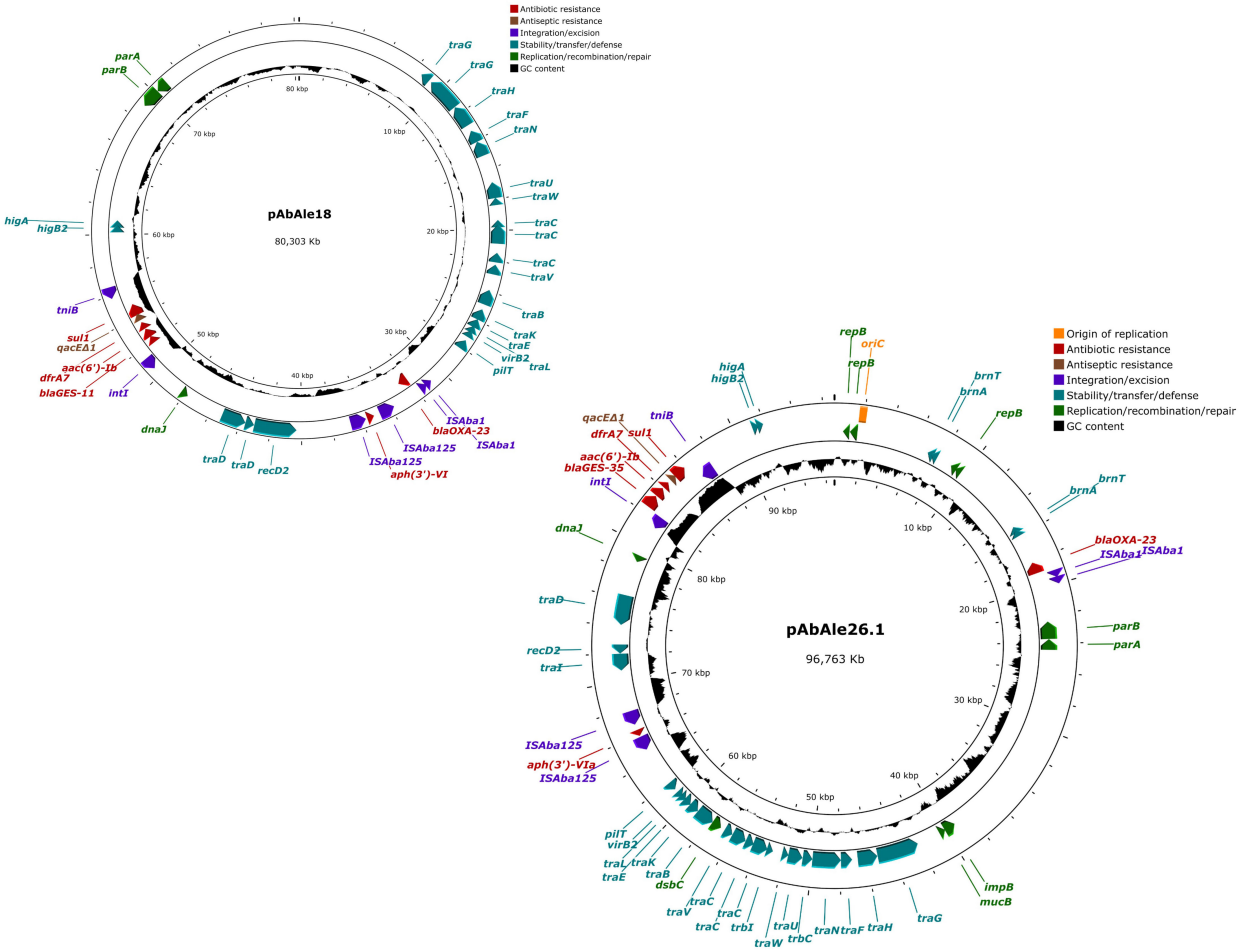
Structure of plasmids pAbAle18 and pAbAle26.1 harboring *bla*_OXA-23_ and *bla*_GES-like_ genes identified by whole genome sequencing in isolates Ale18 and Ale26.

A likely conjugative plasmid of 96.763 Kb (**Figure 4**) together with a mobilizable plasmid of 4.955 Kb (**Supplementary Figure 2**), were identified in isolate Ale26. Many antibiotic resistance genes were located in the conjugative plasmid, including *bla*_OXA-23_ and *bla*_GES-35_ genes, *sul1*, *aph(3’)-VIa*, and two genes that were not previously identified, *aac(6’)-Ib* and *dfrA7*. The rest of the genes (*bla*_ADC-117_, *bla*_OXA-65_, *catB8, mph(E), msr(E), armA, aph(3´)-Ia, ant(3’’)-II, aadA1-pm* and a second copy of *aac(6’)-Ib* and *sul1*) were found in the chromosome. In addition to the antibiotic resistance genes, plasmid pAbAle26.1 also contained the *qacEΔ1* gene.

A single plasmid of 80.303 Kb in size was detected in isolate Ale18 (**Figure 4**). This plasmid was likely conjugative and also harbored *bla*_OXA-23_ and *bla*_GES-11_ genes, together with *aph(3’)-VI*, *aac(6’)-Ib*, *sul1* and *dfrA7* genes, and the quaternary ammonium compound resistance gene *qacEΔ1*. The rest of the antibiotic resistance genes were coded by the chromosome (*bla*_ADC-80_, *bla*_GES-11_, *bla*_OXA-23_, *bla*_OXA-94_ and *ant(3’’)-II*).

In isolate Ale20 a conjugative plasmid of 88.488 Kb in size was detected (**Supplementary Figure 3**). The aminoglycoside resistance gene *aph(3’)-VIa* was identified and located within this element, while the rest of the antibiotic resistance genes were found in the chromosome (*bla*_TEM-1_, *bla*_ADC-25_, *bla*_NDM-1_, *bla*_OXA-23_, *bla*_OXA-66_, *catB8, mph(E), msr(E), armA, aph(3´)-VI, aac(6’)-Ib, ant(3’’)-II, aadA1-pm, sul1* and *aph(3´)-Ia*, discovered by hybrid assembly).

In isolate Ale10 a conjugative plasmid of 70.016 Kb and a mobilizable plasmid of 10.931 Kb (**Supplementary Figure 4**) were identified. All the antibiotic resistance genes were found within the chromosome (*bla*_ADC-143_, *bla*_PER-7_, *bla*_OXA-23_, *bla*_OXA-66_, *catB8, mph(E), msr(E), armA, strA, strB, aph(3´)-Ia, aac(6’)-Ib, ant(3’’)-II, aadA1-pm, sul1* and *sul2*) except the aminoglycoside resistance gene *aph(3’)-VI* which, was also coded by plasmid pAbAle10.1 and newly identified by hybrid assembly. A second copy of the *bla*_OXA-23_ gene was also identified by hybrid assembly. It was remarkable the presence of genes implicated in mobilization such as *mobA* in plasmid pAbAle10.2.

### 3.3 Replicon typing

Replicon typing analysis against *Acinetobacter Plasmid Typing database* showed the presence of Rep_3 family and RepPriCT_1 family replicases R3 -T1, R3 -T4, R3-T13, R3-T14, R3-T15, R3-T60 and RP-T1 (**Table 2**). Large plasmids found in isolates Ale9 and Ale21 contained Rep_3 family replicase R3-T60. Rep_3 family replicases were present in all the isolates except in isolate Ale18. Two replicases were detected in plasmids pAbAle20 and pAbAle21.1 belonging to Ale20 and Ale21, respectively.

### 3.4 Conjugation experiments

Attempts to transfer the likely conjugative plasmids by conjugation experiments were not successful in the described conditions.

## 4 Discussion

Dissemination of antimicrobial resistance genes through mobile genetic elements such as plasmids is of high concern, where horizontal transfer represents one of the most important mechanisms for their dissemination. A number of plasmids ranging in size from 1.3 to 400 Kb have been described in *Acinetobacter* spp. However, plasmids smaller than 20 Kb in size are specially frequent and they are usually mobilizable or non-transmissible (Brovedan et al., 2020; Ghaly et al., 2020). In this work, different results were obtained by S1-PFGE and sequencing experiments. S1-PFGE is a valuable technique for high molecular weight plasmid detection, however, this is limited by the plasmid copy number present in the culture when the method is performed, making it difficult to detect them in agarose gels. For this reason, whole genome sequencing experiments were performed revealing the presence of likely conjugative large size plasmids ranging from 70.01 to 205.6 Kb in all the isolates. These plasmids are of special interest, as they code for multiple antibiotic resistance genes and MGE. Similar findings have been described by other authors (Brovedan et al., 2020).

The most widely distributed plasmids in *Acinetobacter* spp. are those with Aci1(R3-T1) and Aci6 (RP-T1) replicases (Lam et al., 2022). Nevertheless, the plasmids we identified in this work contained Aci6 replicases and not Aci1. RepAci6 plasmids are usually conjugative and more than 20 Kb. They frequently harbor antibiotic resistance genes such as *bla*_NDM_, *bla*_OXA-23_, *aph(3’)-VI*, *sul1*, *sul2*, *mph(E)* and *msr(E)* (Blackwell and Hall, 2019; Brovedan et al., 2020). The majority of plasmids that were less than 20 Kb, belonged to the Rep_3 superfamily group and coded for partition genes, toxin/antitoxin systems, zinc acquisition systems and septicolysin, but not for antibiotic resistance genes, which is in accordance with the literature (Partridge Sally et al., 2018; Brovedan et al., 2020). Co-existence of plasmids belonging to different incompatibility groups in the same cell that may accumulate different antibiotic resistance genes is crucial for the development of highly resistant phenotypes (Blackwell and Hall, 2019).

Replicases from isolates Ale10 and Ale20 could not be identified by the Bertini scheme, but sequencing analysis showed they carried the Aci9 and Aci1, respectively. Furthermore, other replicase groups that had not been described in Bertini’s scheme could be identified by the sequence comparison method, since its database includes a higher number of replicases than the old scheme. However, discrepancies between both methodologies were detected. In isolate Ale9, two replicases were observed by AB-PBRT: Aci1/2 and p2S1, while a single replicase was identified by the system described by Lam et al.: R3-T60. A similar phenomenon was observed in isolates Ale21 and Ale26, where previously identified replicases Aci4, Aci8/6 and p2S1 were not detected by sequence comparison. This may be due to the fact that some contigs could not be completely assembled and, as they may belong to plasmids, what could explain the missing replicases. More experiments are needed to rule out the presence of more plasmids. Furthermore, in some isolates, plasmids that could not be classified by replicon typing were identified, as this method is limited by the sequences included in the database of Lam et al. (2022) , which may indicate that these are replicases not described so far.

The *bla*_NDM-1_ gene was chromosome-borne in isolate Ale20, which is the most common location according to other authors (Fernández-Cuenca et al., 2020; Xanthopoulou et al., 2020), although it has also been reported in non-conjugative but mobilizable plasmids alone or together with the *bla*_OXA-58_ gene (Salloum et al., 2018; Liu et al., 2021). Different locations were identified for the *bla*_PER-7_ gene, which was found in the chromosome and in a 205 Kb plasmid in isolates Ale10 and Ale9, respectively. The *bla*_PER-7_ gene has been described before in plasmids of approximately 200 Kb and 190 Kb in the Arab Emirates and India, respectively, and together with other antibiotic resistance genes such as *strA, strB, armA, mph(E), msr(E), cmlA1, arr-2, sul1*, *sul2* and *tet(B)* (Opazo et al., 2012; Vijayakumar et al., 2022). These plasmids that were identified in India harbored the R3-T60 replicase, matching with the replicases identified in our isolates containing *bla*_PER-7_ gene. In isolates Ale18 and Ale26 *bla*_GES_ and *bla*_OXA-23_ genes were located within plasmids of 80 and 96 Kb in size, respectively. Co-existence of *bla*_GES-11_ and *bla*_OXA-23_ genes have been previously reported in conjugative plasmids of approximately 80 Kb and harboring the replicase Aci6 (RP-T1) in Kuwait (Wibberg et al., 2018). These were, in turn, accompanied by other antibiotic resistance genes such as *aacA4, dfrA7* and *sul1*.

The WGS of the selected isolates allowed us to obtain the complete sequence and map a total of 11 plasmids, identifying at least two plasmids in most isolates. Among them, large plasmids of up to 205.657 Kb with multiple antimicrobial resistance genes were identified, which were also conjugative and mobilizable, facilitating their dissemination to other isolates. Plasmids are of high interest as antibiotic resistance control tools. The importance of studying these elements lies in their utility as genetic markers for antibiotic resistance and virulence factors, and as targets for treatment and development of compounds that can inhibit transfer processes to avoid their dissemination in the environment (Palencia-Gándara et al., 2021).

## Data availability statement

The datasets presented in this study can be found in online repositories. The names of the repository/repositories and accession number(s) can be found in the article.

## Author contributions

SS-U: Investigation, Writing – original draft, Writing – review & editing, Data curation, Formal analysis, Methodology, Software, Validation, Visualization. AO-S: Methodology, Writing – review & editing, Supervision. JR-G: Data curation, Methodology, Writing – review & editing. ME-K: Methodology, Writing – review & editing. SS: Methodology, Writing – review & editing. IA: Funding acquisition, Investigation, Writing – review & editing. LG: Funding acquisition, Investigation, Resources, Supervision, Writing – original draft, Writing – review & editing.
