# Supplementary material for: Plasmid content of carbapenem resistant Acinetobacter baumannii isolates belonging to five International Clones collected from hospitals of Alexandria, Egypt
Source: Front Cell Infect Microbiol. 2024 Jan 9;13:1332736. doi: 10.3389/fcimb.2023.1332736 (PMC10803598; doi:10.3389/fcimb.2023.1332736)
Supplement: Supplementary file 1 [file DataSheet_1.pdf]

Supplementary Material

Plasmid content of carbapenem resistant *Acinetobacter baumannii* isolates belonging to five International Clones collected from hospitals of Alexandria, Egypt

Sandra Sánchez-Urtaza[1], Alain Ocampo-Sosa[2,3*], Jorge Rodríguez-Grande[2], Mohammed A. El-Kholy[4], Sherine M. Shawky[5], Itziar Alkorta[6], Lucia Gallego[1*]

*** Correspondence:**

Lucia Gallego: lucia.gallego@ehu.eus

Alain Ocampo-Sosa: alain.ocampo@scsalud.es

1 Supplementary Figures and Tables

1.1 Supplementary Figures

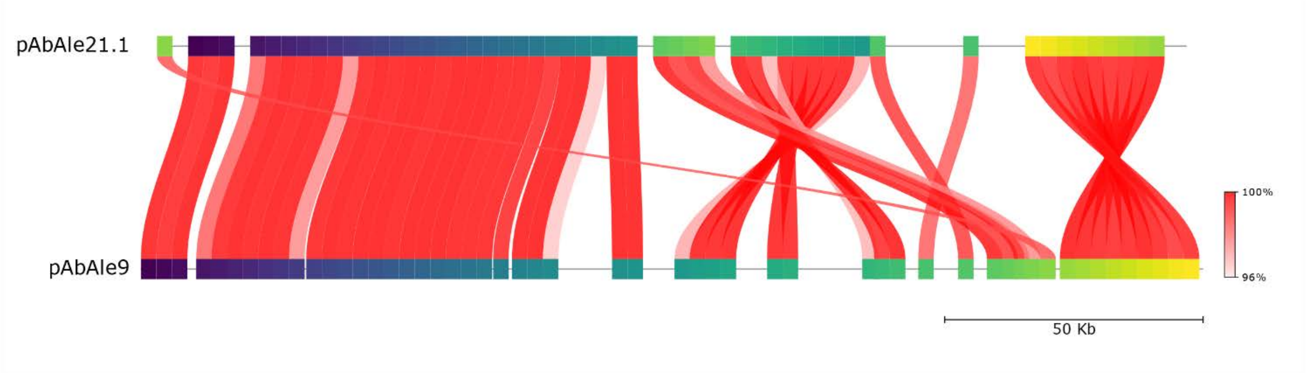

Supplementary Figure 1. Sequence comparison of plasmids pAbAle9 and pAbAle21.1 generated using FastANI 1.3.3 integrated in the online software Proksee.ca. The color bar indicates the percentage of identity between the homologous regions that are connected by segments.

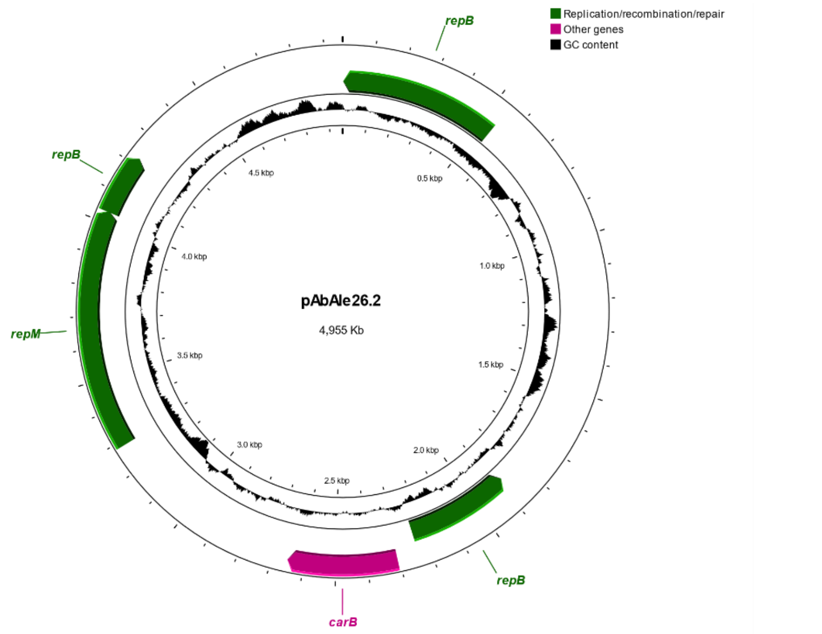

Supplementary Figure 2. Structure of plasmid pAbAle26.2 identified by whole genome sequencing in isolate Ale26.

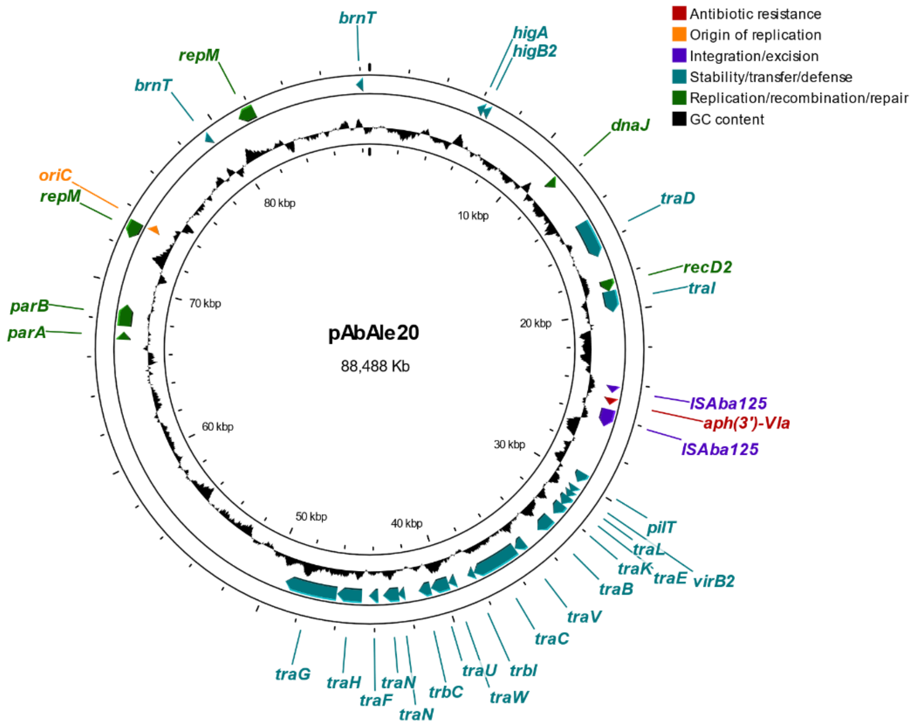

Supplementary Figure 3. Structure of plasmid pAbAle20 identified by whole genome sequencing in isolate Ale20.

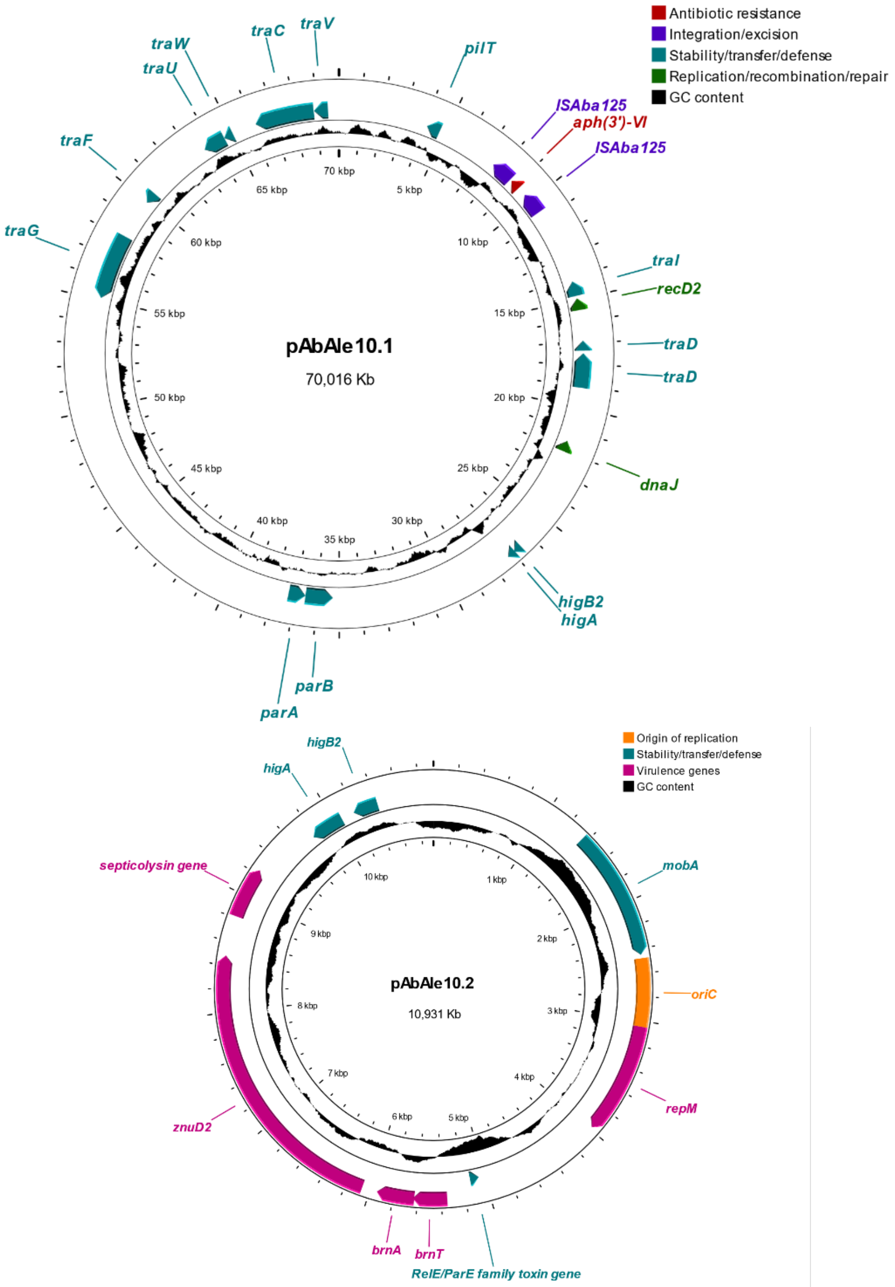

Supplementary Figure 4. Structure of plasmids pAbAle10.1 and pAbAle10.2 identified by whole genome sequencing in isolate Ale10.
